# Sharpbelly Fish Optimization Algorithm: A Bio-Inspired Metaheuristic for Complex Engineering

**DOI:** 10.3390/biomimetics10070445

**Published:** 2025-07-05

**Authors:** Jian Liu, Rong Wang, Yonghong Deng, Xiaona Huang, Zhibin Li

**Affiliations:** 1 School of Computer Engineering, Chengdu Technological University, Chengdu 611730, China; liujian@cdtu.edu.cn; 2 Sichuan Provincial Promotion Center of Digital Transformation, Chengdu 611730, China; hxna1@cdtu.edu.cn (X.H.); lizhibin@cuit.edu.cn (Z.L.); 3 School of Network and Communication Engineering, Chengdu Technological University, Chengdu 611730, China; 4 Key Laboratory of High Performance Scientific Computation, School of Science, Xihua University, Chengdu 610039, China; 5 School of Materials and Environmental Engineering, Chengdu Technological University, Chengdu 611730, China; 6 School of Software Engineering, Chengdu University of Information Technology, Chengdu 610225, China; 7 Xinjiang Technical Institute of Physics & Chemistry, Chinese Academy of Sciences, Urumqi 830011, China

**Keywords:** sharpbelly fish optimizer, swarm intelligence, metaheuristic algorithm, engineering design optimization

## Abstract

This paper introduces a novel bio-inspired metaheuristic algorithm, named the sharpbelly fish optimizer (SFO), inspired by the collective ecological behaviors of the sharpbelly fish. The algorithm integrates four biologically motivated strategies—(1) fitness-driven fast swimming, (2) convergence-guided gathering, (3) stagnation-triggered dispersal, and (4) disturbance-induced escape—which synergistically enhance the balance between global exploration and local exploitation. To assess its performance, the proposed SFO is evaluated on the CEC2022 benchmark suite under various dimensions. The experimental results demonstrate that SFO consistently achieves competitive or superior optimization accuracy and convergence speed compared to seven state-of-the-art metaheuristic algorithms. Furthermore, the algorithm is applied to three classical constrained engineering design problems: pressure vessel, speed reducer, and gear train design. In these applications, SFO exhibits strong robustness and solution quality, validating its potential as a general-purpose optimization tool for complex real-world problems. These findings highlight SFO’s effectiveness in tackling nonlinear, constrained, and multimodal optimization tasks, with promising applicability in diverse engineering scenarios.

## 1. Introduction

With the continuous advancement of science and technology, complex optimization problems have become increasingly prevalent in both scientific research and practical applications. Among them, engineering optimization problems are particularly prominent due to their critical role in improving industrial performance and economic efficiency. These problems often involve fine-tuning process parameters and operational data under complex constraints to maximize productivity or minimize cost while satisfying technical requirements. Solving such problems typically requires the analysis of high-dimensional, nonlinear, and constrained objective functions. Traditional numerical methods often fall short in these scenarios, especially when the feasible region is narrow due to strict inequality constraints and mixed-integer decision variables. To address these challenges, researchers have developed a variety of swarm intelligence algorithms (SIAs), which are a class of metaheuristic approaches inspired by the collective behavior of natural organisms. Swarm intelligence algorithms are particularly suitable for engineering optimization due to their simplicity, flexibility, and global search capabilities [1,2,3]. They can effectively explore complex search spaces and avoid local optima by leveraging stochastic population-based mechanisms. As a result, SIAs have been widely applied in fields such as signal processing, image analysis, medical resource scheduling, very large-scale integration design, and structural engineering optimization [4,5].

Swarm intelligence embodies the collective intelligence exhibited by biological populations through collaboration and interaction. Inspired by such natural behaviors, researchers have proposed a variety of swarm intelligence optimization algorithms. These algorithms are characterized by features such as self-organization, distributed processing, and high adaptability, making them well-suited for solving complex optimization problems. One of the earliest and most representative algorithms is the genetic algorithm (GA) [6], proposed by Holland, which has been widely applied in knapsack problems, image processing, and scheduling tasks. Subsequently, Marco et al. introduced the ant colony optimization (ACO) algorithm [7], inspired by the food-foraging behavior of ants, which is particularly effective for shortest path problems. Kennedy and Eberhart developed the particle swarm optimization (PSO) algorithm [8] by simulating the coordinated flight of bird flocks; PSO guides the search process using both personal and global best solutions and is especially suitable for solving high-dimensional continuous problems. In addition, many other algorithms have drawn inspiration from collective animal behaviors. For example, the bat algorithm (BA) [9] simulates the echolocation-based hunting strategy of bats, mapping the search and exploitation process to prey tracking. The firefly algorithm (FA) [10] is based on the light intensity-driven attraction between fireflies, using brightness (fitness) to guide the search direction. The sparrow search algorithm (SSA) [11], a novel swarm optimization approach inspired by the group wisdom, foraging strategies, and anti-predation behaviors of sparrows, has demonstrated strong performance in both single-objective and multi-objective optimization tasks. The butterfly optimization algorithm (BOA) [12] mimics how butterflies locate food sources and mates using olfactory cues, enabling effective exploration of the solution space.

Considering the increasing complexity of optimization tasks, recent years have witnessed the development of numerous bio-inspired metaheuristic algorithms. These methods have introduced a diverse range of biological inspirations—from algae aggregation to cuckoo mimicry—to improve convergence speed, exploration adaptability, or constraint handling. To provide an overview of this evolving landscape, Table 1 presents a comparative summary of twelve representative algorithms developed over the past five years. The table highlights their design inspirations, major advantages, and known limitations.

Despite their individual strengths, these algorithms still suffer from common challenges, such as premature convergence, insufficient exploration in high-dimensional spaces, or instability under constrained conditions. According to the no free lunch (NFL) theorem (Wolpert and Macready, 1997) [24], no optimization algorithm can perform optimally across all problem domains. This theoretical constraint underscores the ongoing need to develop novel and efficient metaheuristic strategies that are adaptable to specific problem characteristics. Motivated by this, this paper proposes a new metaheuristic algorithm named the sharpbelly fish optimizer (SFO). Inspired by the ecological behaviors of sharpbelly fish—including predation, aggregation, dispersal, and escape—SFO integrates four biologically derived mechanisms to achieve a dynamic balance between global exploration and local exploitation in complex optimization landscapes.

The rest of this paper is organized as follows. Section 2 describes the design principles and search mechanisms of the proposed SFO. Section 3 presents numerical experiments on standard benchmark functions to evaluate the performance of SFO. Section 4 applies the algorithm to several classical engineering design problems to verify its practical effectiveness. Finally, Section 5 concludes the paper and discusses potential directions for future research.

## 2. The Sharpbelly Fish Optimizer (SFO)

### 2.1. Inspiration

**Figure 1:**
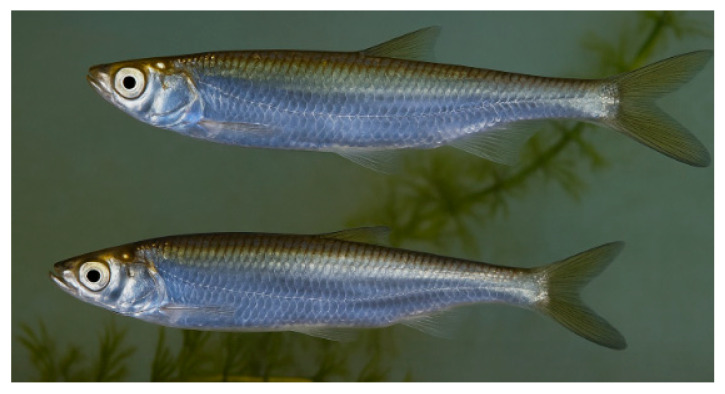
Sharpbelly fish.

The sharpbelly (Hemiculter leucisculus, Basilesky, 1855) [25,26,27] is a small, fast-swimming freshwater and brackish-water fish belonging to the family Xenocyprididae, as shown in Figure 1. It is widely distributed in East Asia, including mainland China and Korea, and has been introduced to various countries such as Iran and Afghanistan, where it has demonstrated strong ecological adaptability and invasiveness. This species primarily inhabits low-elevation freshwater bodies, such as streams, lakes, and reservoirs, and typically resides in the upper to middle water column, at depths ranging from 0 to 10 m. The optimal environmental conditions for the sharpbelly include a pH of approximately 7.0, water hardness around 15 DH, and a temperature range of 18–22 °C (64–72 °F). Behaviorally, the sharpbelly is characterized by its active, agile, and highly sensitive nature. It is a schooling fish often found swimming in shallow waters near the surface, where it exhibits predatory behavior, such as chasing flying insects above the water. The species shows a preference for hovering near aquatic vegetation and open gaps in submerged plant beds in search of food. Sharpbellies are omnivorous, feeding on a diverse range of items including zooplankton, aquatic insects, algae, crustaceans, and plant debris. They are known to deposit adhesive eggs on aquatic plants during spawning [28]. Due to their strong reproductive capacity and environmental adaptability, they are considered ecologically resilient, often dominating in invaded habitats, and outcompeting native species.

**Figure 2:**
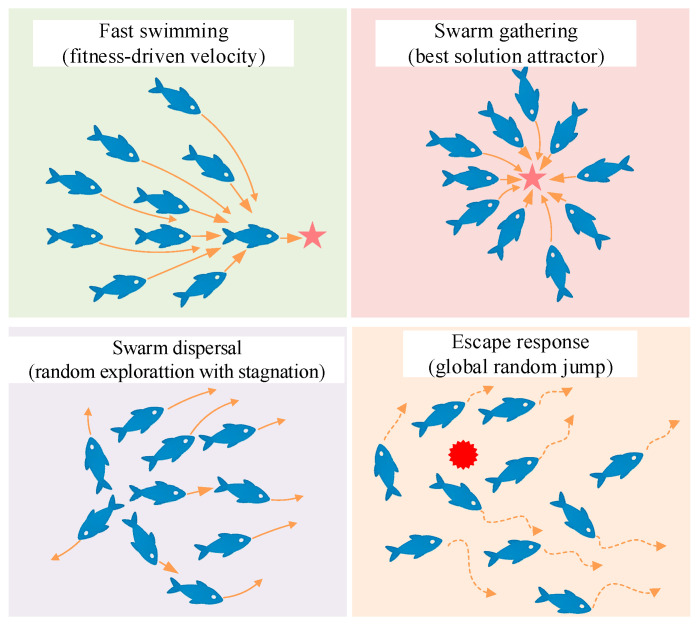
Four core behaviors in the Sharpbelly fish.

The sharpbelly fish exhibits a range of instinctive group behaviors in its natural habitat that offers rich inspiration for designing swarm-based optimization strategies. These behaviors include active predation near the surface, group aggregation, high sensitivity to environmental stimuli, and evasive maneuvers under disturbance. Such adaptive traits are highly aligned with the fundamental principles of intelligent search and collective decision-making in optimization algorithms. Inspired by these ecological characteristics, this paper proposes a novel metaheuristic algorithm named the sharpbelly fish optimizer (SFO). As illustrated in Figure 2, four core behavioral patterns: fast swimming, swarm gathering, dispersal, and escape response, serve as the foundation for the algorithmic design of SFO.

Comparison with other fish-inspired algorithms: to clarify the distinctions between the proposed SFO and other biologically inspired algorithms such as Bitterling Fish Optimization (BFO) and Pufferfish Optimization Algorithm (PFO), a comparison is provided in terms of biological inspiration, search mechanism, and structural design.

(1) The biological origin of SFO is derived from the foraging and evasion behaviors of Hemiculter leucisculus (sharpbelly fish), which exhibit cooperative swimming, opportunistic aggregation, and rapid dispersal under threats. In contrast, BFO is inspired by the reproductive behavior of bitterling fish, focusing on host mussel selection and spawning strategy. PFO models the self-defensive inflation behavior and localized movement of pufferfish. Thus, while BFO and PFO focus on reproductive or defensive behaviors, SFO emphasizes swarm intelligence and motion-driven adaptation.

(2) Behavioral modeling and update dynamics: The SFO algorithm integrates four core strategies: swim (directional movement), gather (group-based attraction), disturb (randomized noise injection), and disperse (diversity enhancement under stagnation), forming a balanced exploration–exploitation mechanism. BFO, in contrast, relies on discrete egg-laying decisions and lacks continuous positional updates, limiting its performance in high-dimensional continuous domains. PFO utilizes radial motion and repulsion behavior but lacks collective intelligence modeling or stagnation escape strategies.

(3) Mechanism novelty and algorithm robustness: SFO introduces an explicit stagnation detection mechanism based on the Ts threshold, triggering global dispersal to prevent premature convergence. Additionally, the velocity-based update model in SFO allows dynamic adaptation to landscape complexity. These mechanisms are absent or simplified in BFO and PFO, making SFO more suitable for solving complex numerical optimization problems.

In summary, although all three algorithms are inspired by aquatic species, SFO distinguishes itself through a motion-coordination framework, behavioral diversity, and stagnation-driven adaptivity, which collectively contribute to its superior convergence stability and robustness across various benchmark functions.

### 2.2. The Mathematical Model of SFO

#### 2.2.1. Population Initialization

In the proposed SFO algorithm, each individual sharpbelly fish represents a potential candidate solution within the search space of a given optimization problem. The position of each fish is modeled as a point in a *D*-dimensional continuous space, where *D* denotes the number of decision variables. The algorithm starts by initializing a population of *N* individuals, whose positions are randomly distributed within the defined boundaries. The initial population matrix X_0_ ∈ *R^N^*^×*D*^ is defined as follows:

(1) $$X^{0}=\mathrm{rand}(N,D)\cdot (ub-lb)+lb$$

where *X*^0^ is the initial position matrix at iteration *t* = 0, rand(*N*, *D*) is a uniformly distributed random matrix with values in [0, 1], and *ub*, *lb* ∈ *R^D^* are the upper and lower bounds of the search space.

This initialization ensures that each fish begins its search from a feasible location within the problem domain. The fitness of each individual is evaluated using an objective function, with results stored in the fitness vector *F^t^* ∈ *R^N^*^×1^.

(2) $$F^{t}={\left[f(X_{1}^{t}),f(X_{2}^{t}),\cdots ,f(X_{N}^{t})\right]}^{T}$$

where *f*(·) represents the fitness function.

#### 2.2.2. Swimming and Gathering

To model the innate tendency of sharpbelly fish to seek nutrient-rich zones, a basic swimming behavior is implemented. Each fish moves in the direction of the global best solution, with a speed inversely proportional to its fitness value. This mechanism reflects the ecological observation that weaker individuals tend to exhibit more exploratory movement.

(3) $$V_{i}^{swim}=s_{i}^{t}\cdot rand()\cdot (X_{best}^{t}-X_{i}^{t})$$

(4) $$s_{i}^{t}=1-\frac{f(X_{i}^{t})}{\max_{j}f(X_{j}^{t})+\varepsilon }$$

where $V_{i}^{swim}$ represents the swimming velocity vector of the *i*-th individual, and the swim speed $s_{i}^{t}$ determined by the relative fitness of the individual compared to the worst solution in the population. Specifically, max*_j_ f*($X_{j}^{t}$) represents the worst fitness value at iteration *t*, which is used to normalize the swimming speed of each individual. The constant *ε* is a small positive number to prevent division by zero.

Simultaneously, all fish are influenced by a social cohesion mechanism, prompting them to gather near the current global optimum:

(5) $$V_{i}^{gather}=\alpha \cdot rand()\cdot (X_{best}^{t}-X_{i}^{t})$$

where $V_{i}^{gather}$ represents the gathering velocity of the *i*-th individual, which drives it toward the global best position $X_{best}^{t}$. The parameter α ∈ (0, 1) controls the social attraction strength toward the global best position, facilitating exploitation regardless of fitness.

#### 2.2.3. Escape Behavior

In nature, sharpbelly fish react strongly to disturbances in their habitat by rapidly changing direction. To emulate this behavior, a stochastic escape mechanism is triggered with a probability *Pf*, modeled by Gaussian-distributed random motion.

(6) $$V_{i}^{disturb}=\left\{\begin{array}{l} \delta \cdot N(0,1),ifrand<Pf\\ 0,\begin{array}{cccc} & otherwise & & \end{array} \end{array}\right.$$

where $V_{i}^{disturb}$ denotes the disturbance velocity vector of the *i*-th individual. *δ* controls the disturbance magnitude and *N*(0, 1) is a standard normal vector. *Pf* ∈ [0, 1] is the disturbance probability, which controls how frequently individuals are subjected to random perturbation. A higher value of *Pf* promotes exploration, while a lower value maintains stability.

#### 2.2.4. Dispersal Due to Stagnation

When the algorithm exhibits stagnation—defined as a lack of improvement over a fixed number of iterations *T_s_*—a dispersal mechanism is activated to help individuals escape local optima. In this mode, affected individuals randomly explore the space within a bounded radius:

(7) $$V_{i}^{disperse}=\left\{\begin{array}{l} \gamma \cdot (2\cdot rand()-1)(ub-lb),ifc_{no\_improve}\geq T_{s}\\ 0,\begin{array}{cccc} & otherwise & & \end{array} \end{array}\right.$$

where $V_{i}^{disperse}$ represents the dispersal velocity of the *i*-th individual, which is introduced to prevent stagnation. When the individual has not improved its fitness for *T_s_* consecutive iterations, it is relocated within the search space using a random vector scaled by *γ*. The parameter *c*_no_improve_ monitors this stagnation period. Here, *γ* ∈ [0, 1] determines the dispersal rang.

#### 2.2.5. Position Update

The final velocity of each fish is the sum of the active behavior components:

(8) $$V_{i}^{t}=V_{i}^{swim}+V_{i}^{gather}+V_{i}^{disturb}+V_{i}^{disperse}$$

Each fish updates its position based on the resulting velocity vector, according to the following rule:

(9) $$X_{i}^{t+1}=X_{i}^{t}+V_{i}^{t}$$

To ensure feasibility, each updated position is projected onto the feasible domain:

(10) $$X_{i}^{t+1}=\min(\max(X_{i}^{t+1},lb),ub)$$

This projection operation ensures that each dimension of the updated position vector lies within the predefined lower and upper bounds, thereby maintaining feasibility throughout the search process.

At the end of each iteration, the best individual is updated as follows:

(11) $$X_{best}^{t+1}=\left\{\begin{array}{l} X_{i}^{t+1},iff(X_{i}^{t+1})<f(X_{best}^{t})\\ X_{best}^{t},\begin{array}{cccc} & otherwise & & \end{array} \end{array}\right.$$

**Figure 3:**
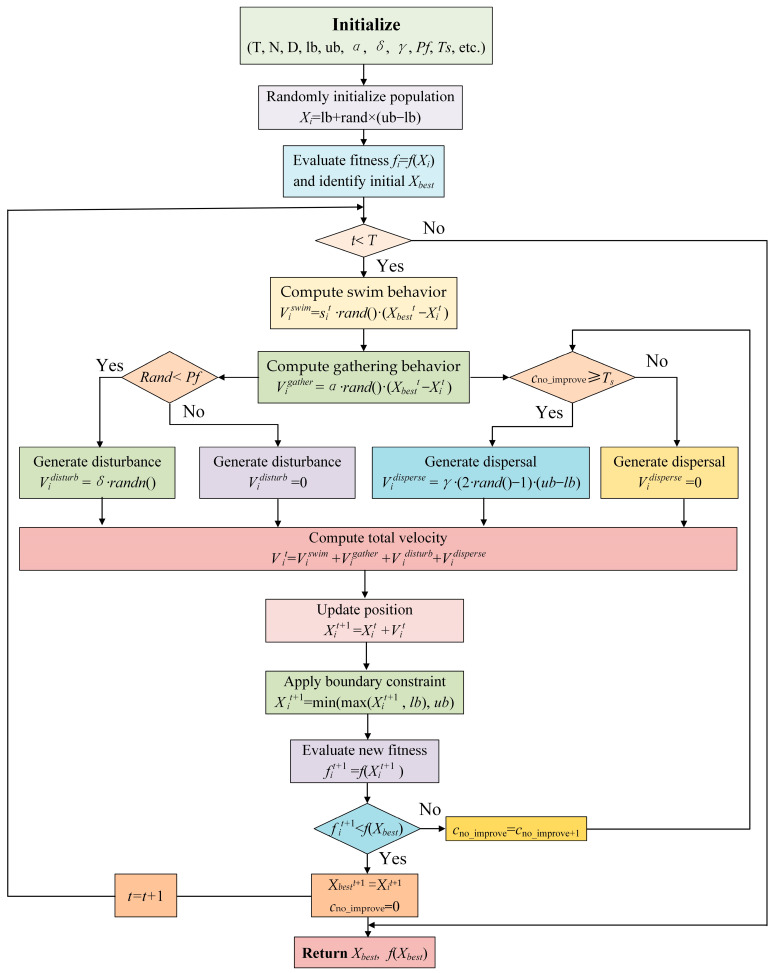
The detailed workflow of SFO.

To summarize, in SFO, the optimization process is driven by the integration of four biologically inspired behaviors—swimming, gathering, escape, and dispersal—which collectively determine the search dynamics of each fish. These behaviors are mathematically modeled to guide the individuals toward high-quality solutions while maintaining population diversity. The velocity-based update mechanism enables the algorithm to balance global exploration and local exploitation effectively. Once the position is updated and feasibility is ensured, the global best solution is retained to guide subsequent search. This iterative process continues until the termination criterion is met. The detailed workflow of SFO is illustrated in Figure 3, where each step corresponds to the key equations and mechanisms in Algorithm 1. Please note that the complete source code for the SFO is publicly available at https://github.com/DYHuestc/SFO-project (accessed on 2 July 2025).

### 2.3. Computational Complexity

The computational complexity of the proposed SFO is analyzed based on its main procedures, including population initialization, fitness evaluation, and position update. Let *N* denote the population size, *D* the problem dimension, and *T* the maximum number of iterations. The initialization process involves generating *N* individuals in a *D*-dimensional search space, yielding a complexity of *O*(*N* × *D*). During each iteration, the algorithm evaluates the fitness of all individuals, which requires *O*(*N*) operations. Additionally, the velocity update consists of four behavior mechanisms—swimming, gathering, escape, and dispersal—each involving vector operations with complexity *O*(*D*) per individual. Therefore, the overall position update in one iteration incurs a cost of *O*(*N* × *D*), and across TTT iterations, the total becomes *O*(*N* × *T* × *D*). The global best solution is updated at each iteration by comparing fitness values, which adds a negligible cost of *O*(*N*) per iteration. In total, the computational complexity of the SFO algorithm is *O*(*N* × *T* × *D*). This indicates that the proposed algorithm scales linearly with respect to the population size, the number of iterations, and the dimensionality of the problem, making it suitable for solving high-dimensional optimization problems efficiently.
**Algorithm 1** SFOBegin1.**Initialize** (T, N, D, lb, ub, *α*, *δ*, *γ*, *Pf*, *Ts*, etc.)2.Randomly initialize population *X_i_* = lb + rand × (ub−lb)3.Evaluate fitness *f_i_* = *f*(*X_i_*), and identify initial *X_best_*4.t = 0; stagnation_count = 05.**for** *t* = 1 **to** *T*6.   **for**
*i* = 1 **to**
*N*7.     Compute swim behavior:* $V_{i}^{swim}$*= $s_{i}^{t}$*·rand*()·($X_{best}^{t}$−$X_{i}^{t}$)8.     Compute gathering behavior: $V_{i}^{gather}$ = *α·rand*()·($X_{best}^{t}$−$X_{i}^{t}$)9.    **if** rand < *Pf*10.     Generate disturbance: $V_{i}^{disturb}$ = *δ·randn*()11.    **else**12.     $V_{i}^{disturb}$ = 013.    **end if**14.    **if**
*c*_no_improve_ ≥ *T_s_*15.     Generate dispersal:*$V_{i}^{disperse}$*= *γ·*(2*·rand*()−1)*·*(ub−lb)16.    **else**17.     $V_{i}^{disperse}$ = 018.    **end if**19.    Compute total velocity: $V_{i}^{t}$ = $V_{i}^{swim}$ + $V_{i}^{gather}$ + $V_{i}^{disturb}$ + $V_{i}^{disperse}$20.    Update position: $X_{i}^{t+1}$ = $X_{i}^{t}$
*+ $V_{i}^{t}$*21.    Apply boundary constraint: $X_{i}^{t+1}$ = min(max($X_{i}^{t+1}$, lb), ub)22.    Evaluate new fitness $f_{i}^{t+1}$ = *f*($X_{i}^{t+1}$)23.    **if** $f_{i}^{t+1}$ < *f*(*X_best_*)24.         $X_{best}^{t+1}$ = $X_{i}^{t+1}$*; c*_no_improve_ = 025.     **else**26.       *c*_no_improve_ = *c*_no_improve_ + 127.    **end if**28.     **end for**29.**end for**30.**Return** *X_best_, f*(*X_best_*)

## 3. Experimental Results and Analysis

### 3.1. Experimental Setting

#### 3.1.1. Benchmark Test Functions

Benchmark test functions provide a standardized and rigorous framework for evaluating the effectiveness and robustness of optimization algorithms. In this study, the CEC2022 benchmark suite is employed to assess the performance of the proposed SFO algorithm. Specifically, the test functions are configured under two dimensional settings: 10 and 20. As the problem dimensionality increases, the landscape complexity typically grows due to the presence of more local optima, posing greater challenges for global search algorithms. The CEC2022 suite offers a comprehensive set of benchmark functions encompassing various difficulty levels and landscape characteristics. These include unimodal functions for testing exploitation capability, multimodal functions for evaluating the ability to escape local optima, as well as hybrid and composition functions that simulate complex real-world optimization scenarios. Such a diversified set of benchmark problems ensures a thorough evaluation of the algorithm’s global exploration and local exploitation performance under varying search conditions. Table 2 summarizes the CEC2022 benchmark functions adopted in this study. These functions span unimodal, hybrid, and composition types, providing diverse and representative challenges for evaluating the algorithm’s robustness, stability, and adaptability.

#### 3.1.2. Competitor Algorithms and Parameters Setting

To evaluate the optimization performance of the proposed SFO, several widely recognized metaheuristic algorithms were selected for comparative testing. These algorithms have demonstrated competitive results in various benchmark and engineering optimization problems. A brief overview of each algorithm is presented as follows:

Particle Swarm Optimization (PSO) [29]: a population-based algorithm inspired by the social behavior of bird flocking, where individuals (particles) adjust their trajectories based on their own experience and that of their neighbors to converge toward promising regions.

Ant Colony Optimization (ACO) [30]: inspired by the foraging behavior of ants, ACO uses pheromone trails and probabilistic decision rules to find optimal paths through combinatorial spaces, making it effective for discrete optimization problems.

Genetic Algorithm (GA) [31]: based on the principles of natural selection and genetics, GA operates through selection, crossover, and mutation operators to evolve a population of solutions toward optimality over generations.

Grey Wolf Optimizer (GWO) [32]: mimics the leadership hierarchy and hunting mechanism of grey wolves in nature, where individuals update positions based on the guidance of alpha, beta, and delta wolves to balance exploration and exploitation.

Sparrow Search Algorithm (SSA) [33]: simulates the foraging and anti-predation behavior of sparrows, dividing individuals into discoverers and joiners to coordinate global search and local refinement effectively.

Bat Algorithm (BA) [34]: inspired by echolocation behavior in bats, this algorithm uses frequency tuning and loudness adjustment to guide individuals toward optimal solutions.

Whale Optimization Algorithm (WOA) [35]: emulates the spiral bubble-net hunting strategy of humpback whales, incorporating both encircling prey and stochastic search components to perform global exploration and local exploitation.

The control parameters of these algorithms were set according to commonly recommended values in the literature and are summarized in Table 3. To ensure a fair comparison, all algorithms use parameter settings either recommended in the original papers or commonly adopted in benchmark studies, without additional fine-tuning. For fairness, all algorithms shared the same experimental conditions: the population size *n* was set to 30, and the maximum number of iterations *T* was fixed at 1000. Each algorithm was independently executed 30 times to account for stochastic variability, and all relevant performance metrics were recorded.

### 3.2. Results Analysis

**Figure 4:**
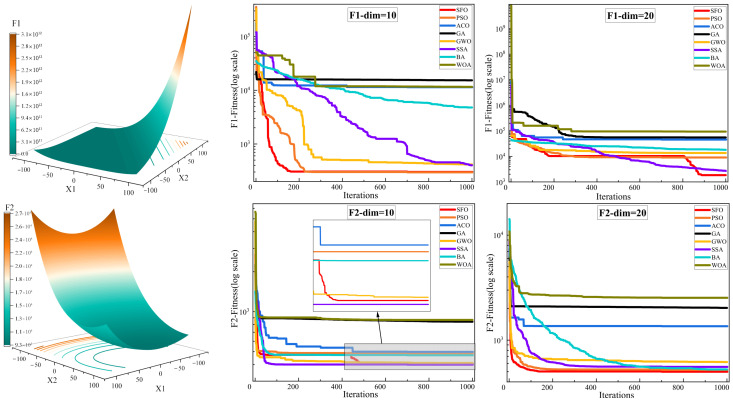

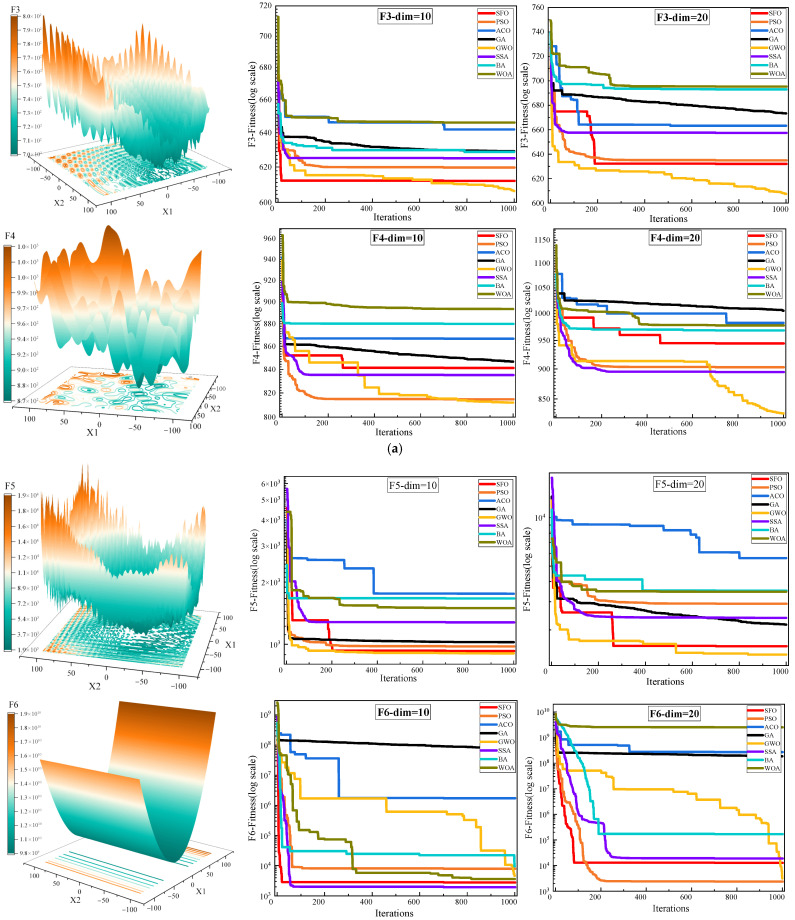

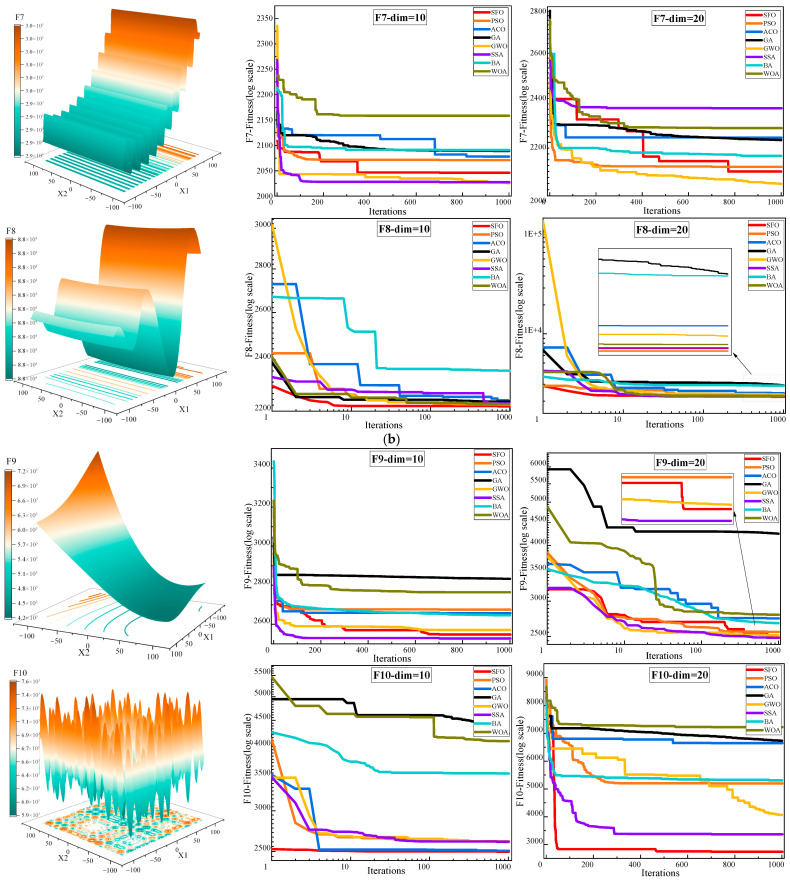

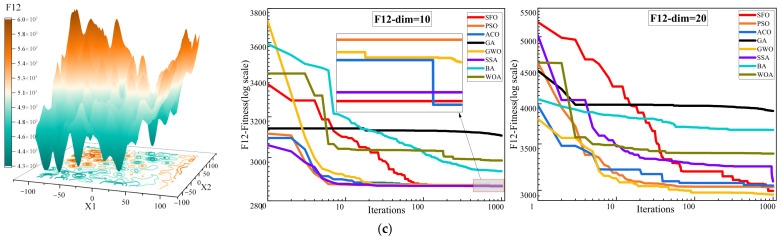
Convergence behavior analysis of various algorithms on the CEC2022 benchmark. (**a**) F1–F4; (**b**) F5–F8; and (**c**) F9–F12.

**Figure 5:**
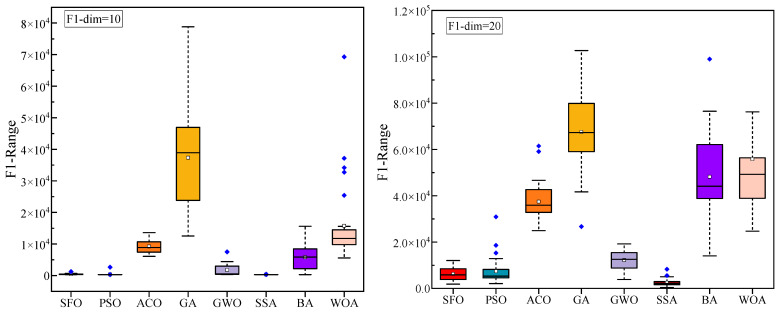

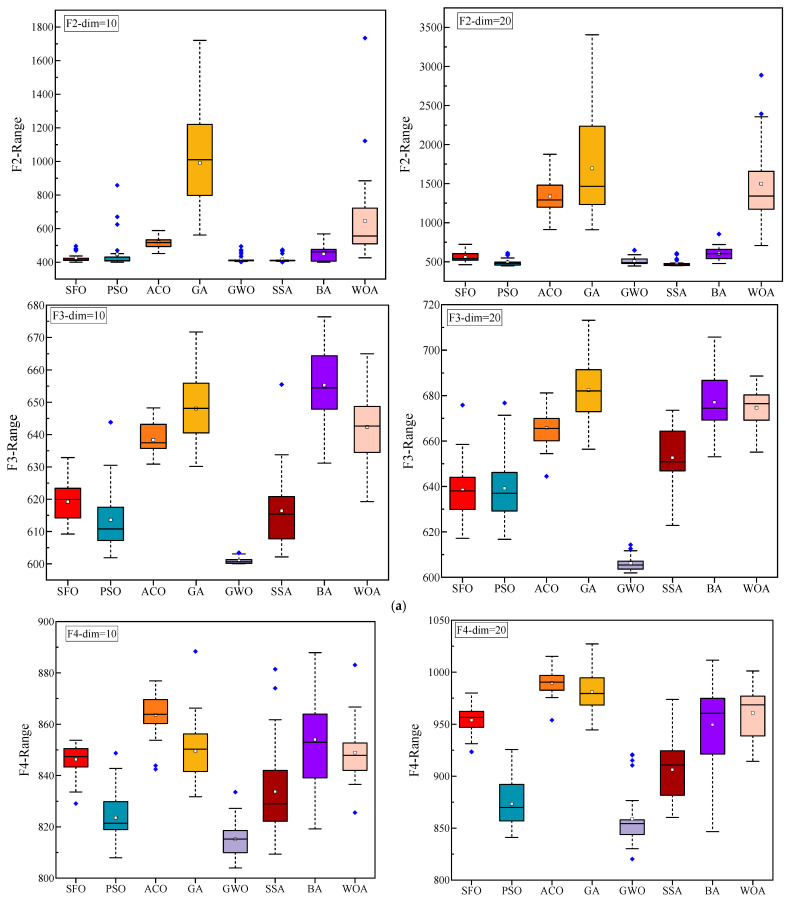

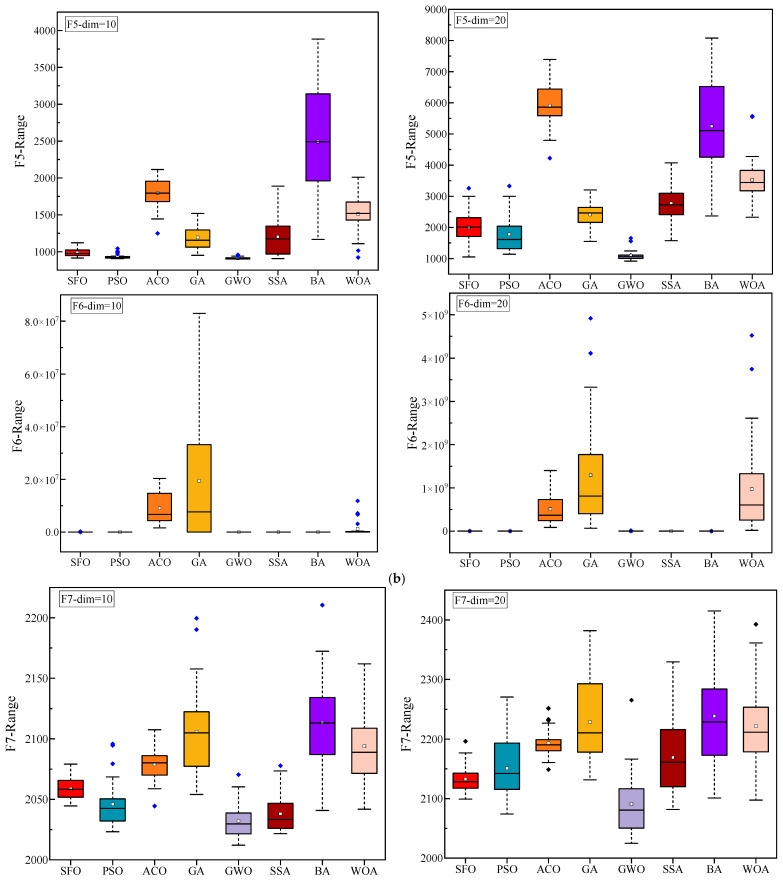

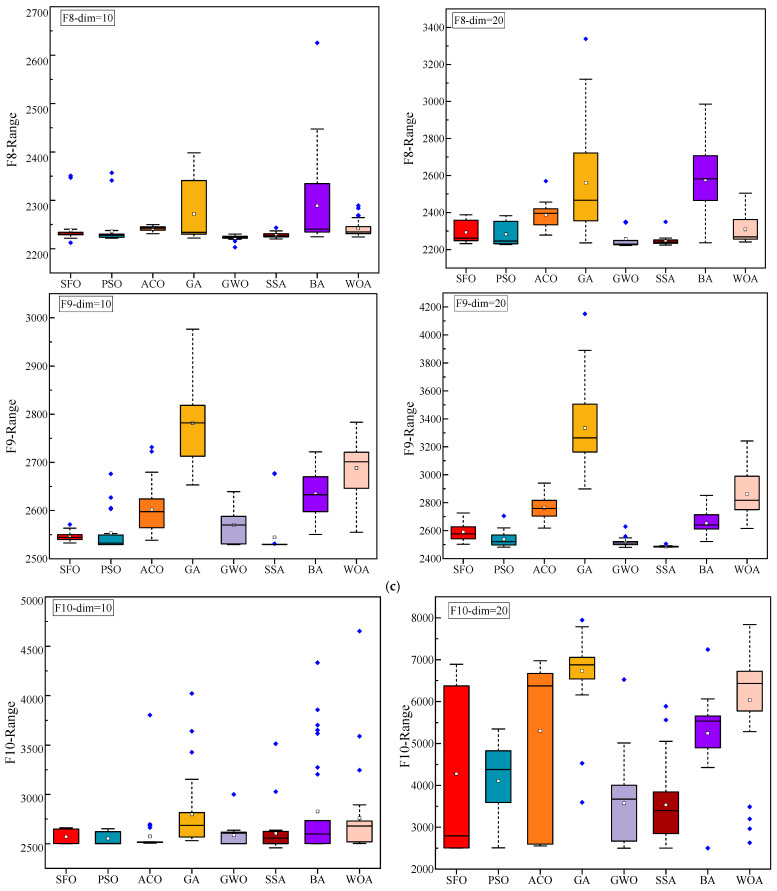

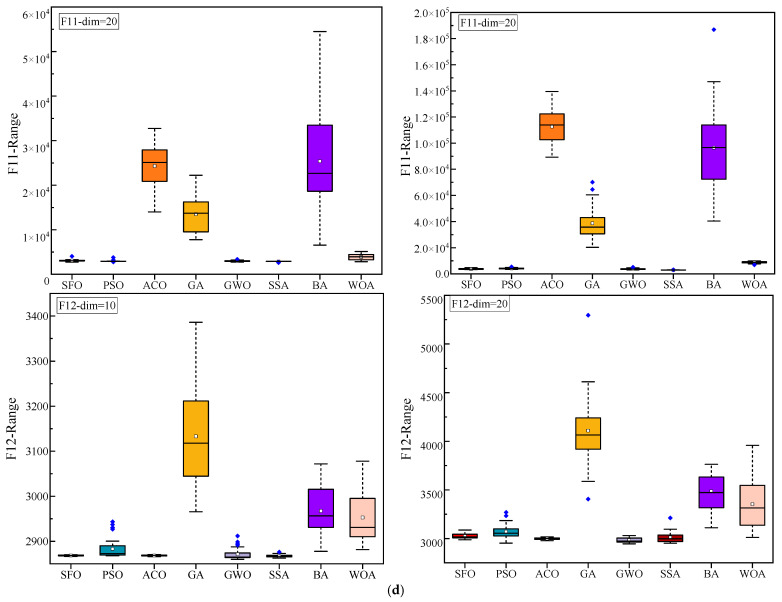
Statistical performance variation (boxplots) of algorithms on CEC2022 functions. (**a**) F1–F3; (**b**) F4–F6; (**c**) F7–F9; and (**d**) F10–F12.

Figure 4 illustrates the convergence curves of various algorithms on the CEC2022 benchmark functions under different dimensional settings (Dim = 10 and Dim = 20), providing an intuitive comparison of their optimization speed and convergence stability. Figure 5 presents the boxplot distributions of the optimization results obtained from 30 independent runs for each algorithm, highlighting the robustness and variability of their performance across the CEC2022 test suite. Table 4 reports the detailed numerical results of the compared algorithms, including the maximum, mean, and standard deviation values over 30 independent runs under both Dim = 10 and Dim = 20. These statistical indicators comprehensively reflect the optimization accuracy, consistency, and reliability of each method across all test functions.

To comprehensively evaluate the optimization performance of the proposed SFO, Figure 4 and Figure 5, along with Table 3, present a detailed comparison against seven well-established metaheuristic algorithms across the CEC2022 benchmark functions under two dimensional settings (Dim = 10 and Dim = 20).

Figure 4 illustrates the convergence behavior of all competing algorithms. It is evident that SFO consistently achieves faster convergence rates and lower objective function values across most benchmark functions, particularly in complex landscapes such as F1, F6, and F11, demonstrating strong exploitation capability and convergence efficiency. Especially in high-dimensional scenarios (Dim = 20), SFO maintains a clear advantage in both convergence speed and stability, avoiding the premature stagnation observed in algorithms such as GA and ACO. However, it is worth noting that in several test functions, other algorithms achieve better convergence performance than SFO. For example, SSA outperforms SFO on functions F2, F6, F7, F9, and F11, likely due to its dynamic foraging and vigilance behavior that adapts well to multimodal or discontinuous landscapes. Similarly, GWO achieves superior results on F3–F5, F7, and F12, benefiting from its hierarchical leadership model that guides the population efficiently in functions with narrow valleys or deceptive basins. PSO, known for its rapid convergence, outperforms SFO on F4 and F6 (Dim = 20), while ACO shows better performance on F12 (Dim = 10) due to its memory-based pheromone-guided search. These results indicate that while SFO performs competitively across most scenarios, other algorithms can surpass it in specific problem classes with favorable structural alignment.

Figure 5 displays the boxplot distributions of the optimization results over 30 independent runs. The compact interquartile range and relatively low dispersion of SFO results indicate its robust performance and superior stability in most cases. In functions such as F2, F7, and F12, SFO achieves lower medians and presents fewer outliers, highlighting its reliability under stochastic conditions. Nevertheless, in several specific functions, other algorithms demonstrate better statistical distributions than SFO: In F2 (both dimensions), SSA slightly outperforms SFO, likely due to its effective role-switching mechanism between discoverers and followers, which provides stronger exploration in early iterations. In F3 and F4, GWO and PSO show improved consistency and median values. These advantages are attributed to their structured exploitation strategies and adaptive velocity updates, respectively. For F6 (Dim = 20), PSO exhibits better performance, benefiting from its inertia-controlled exploration behavior that facilitates early convergence in rugged search spaces. In F12, SFO is marginally outperformed by ACO (Dim = 10) and GWO (Dim = 20), both of which incorporate pheromone reinforcement or hierarchical guidance, offering improved stability in composite functions. These findings suggest that while SFO performs competitively across most test cases, its performance can be slightly surpassed by algorithms whose internal dynamics are better aligned with the characteristics of certain benchmark functions.

Table 4 provides the statistical metrics (maximum, mean, and standard deviation) of the optimization results over 30 independent runs. SFO frequently ranks among the top performers in terms of both average accuracy and variance control. Compared to classical algorithms like GA and BA, which exhibit high variability and sensitivity to initialization, SFO maintains lower standard deviation values and better mean convergence accuracy, reflecting its strong repeatability and robustness across diverse landscapes. However, it is noteworthy that in some specific benchmark functions, other algorithms exhibit superior performance over SFO: F2 (Dim = 10, 20): SSA achieves better convergence than SFO. This can be attributed to the adaptive step-size and role-based role division strategy in SSA, which enhances its local search ability in unimodal functions like F2, especially under low and moderate dimensions. F3 and F5 (Dim = 10, 20): GWO surpasses SFO in convergence performance. The Grey Wolf Optimizer benefits from its balanced hierarchical leadership structure, which enables efficient navigation in complex bowl-shaped function landscapes and contributes to its superior stability and global search capability on these multimodal functions. F4 (Dim = 10, 20): SSA, GWO, and PSO all outperform SFO. F4 is a hybrid composition function with significant landscape shifts. PSO’s velocity-position memory mechanism and SSA’s local adaptive behavior may help avoid stagnation more effectively than SFO in such composite landscapes. F6 (Dim = 20): PSO slightly surpasses SFO in mean performance. This is likely due to PSO’s strong convergence tendency, which in this case provides an advantage in a more rugged landscape where fine-tuning around global optima is essential. F7 (Dim = 10, 20): GWO and SSA outperform SFO. Their social hierarchy (GWO) and vigilance-based dynamic search (SSA) enhance exploration in noisy or discontinuous regions represented by F7. F9 (Dim = 10, 20): SSA again achieves better accuracy than SFO, which may be due to its predator-prey inspired escape mechanism, allowing efficient exit from deceptive local minima in non-separable problems. F11 (Dim = 20): SSA slightly outperforms SFO. F11 is known for deceptive local optima; SSA’s local–global trade-off mechanisms may help in this context. F12 (Dim = 10): ACO achieves better performance than SFO. Ant Colony Optimization’s path memory and pheromone update strategies may better adapt to the intricate structures in this rotated hybrid function. F12 (Dim = 20): GWO shows superior convergence. This suggests that in high-dimensional and highly irregular landscapes, GWO’s balance between exploration and exploitation may slightly outperform SFO’s fish behavior modeling.

The results indicate that SFO demonstrates competitive and well-balanced performance across convergence speed, solution quality, and robustness. It maintains superiority or near-top rankings in most benchmark functions and dimensional settings. However, the observation that other algorithms occasionally outperform SFO on specific functions aligns with the No-Free-Lunch (NFL) theorem, which states that no optimization algorithm can be universally superior across all problem domains. The varied landscape characteristics of benchmark functions naturally favor different algorithmic behaviors, highlighting the importance of diversity in algorithm design and the potential of hybridization or problem-specific enhancements for future improvements of SFO.

## 4. Representative Engineering Design Cases

In this section, the proposed SFO is applied to three classical engineering design problems to evaluate its optimization performance in practical scenarios. These problems include the pressure vessel design problem (PVD) [36], the speed reducer design problem [37], and the gear train design problem [38], which are widely recognized benchmarks in the field of structural and mechanical optimization. These design problems are characterized by nonlinear, constrained, and multi-variable objective functions, making them suitable for testing the global search capabilities and convergence robustness of metaheuristic algorithms. For each case, the mathematical formulation, design constraints, and variable bounds are retained in accordance with the respective literature to ensure comparability of results. The effectiveness of SFO in solving these problems is assessed based on solution quality, convergence speed, and constraint-handling performance, and is compared with existing optimization approaches.

### 4.1. Pressure Vessel Design Problem

**Figure 6:**
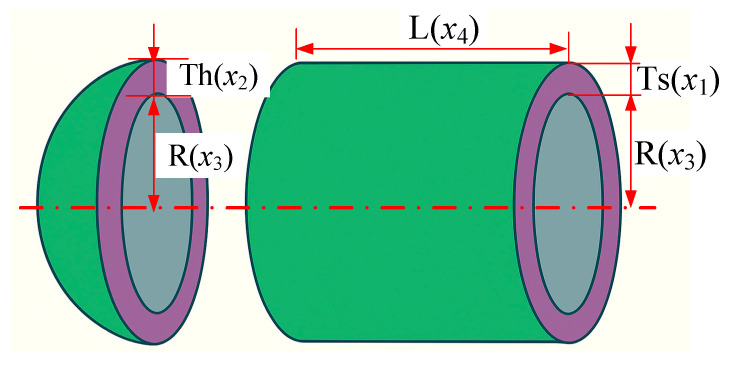
Schematic diagram of the pressure vessel design problem.

The pressure vessel design problem is a widely cited benchmark in structural engineering optimization. The design consists of a cylindrical vessel capped with two hemispherical heads. The primary objective is to minimize the total fabrication cost, which comprises the material cost, forming cost, and welding cost. To achieve this, the design must determine four key variables: the thickness of the shell and head (denoted as Ts = x_1_ and Th = x_2_, respectively), the inner radius of the vessel (R = x_3_), and the length of the cylindrical section excluding the head (L = x_4_), as illustrated in Figure 6.

The mathematical formulation of the optimization problem is as follows:

(12) $$\begin{array}{c} \mathrm{Consider}:x=[\begin{array}{cccc} x_{1} & x_{2} & x_{3} & x_{4} \end{array}]\\ \mathrm{Minimize}:f(x)=0.6224x_{1}x_{3}x_{4}+1.7781x_{2}x_{3}^{2}+3.1661x_{1}^{2}x_{4}+19.84x_{1}^{2}x_{3}\\ \mathrm{Subject}\;\mathrm{to}:\left\{\begin{array}{l} g_{1}(x)=-x_{1}+0.0193x_{3}\leq 0\\ g_{2}(x)=-x_{2}+0.00954x_{3}\leq 0\\ g_{3}(x)=-\pi x_{3}^{2}x_{4}-\frac{4}{3}\pi x_{3}^{3}+1,296,000\leq 0\\ g_{4}(x)=x_{4}-240\leq 0 \end{array}\right.\\ \;\mathrm{Bounds}:\left\{\begin{array}{l} 0.0625\leq x_{1},x_{2}\leq 99\\ 10\leq x_{3},x_{4}\leq 200 \end{array}\right. \end{array}$$

To evaluate the performance of the proposed SFO on engineering design tasks, a benchmark test was conducted on the pressure vessel design problem. Seven algorithms, including SFO, PSO, ACO, GWO, SSA, BA, and WOA, were tested independently over 50 runs.

**Figure 7:**
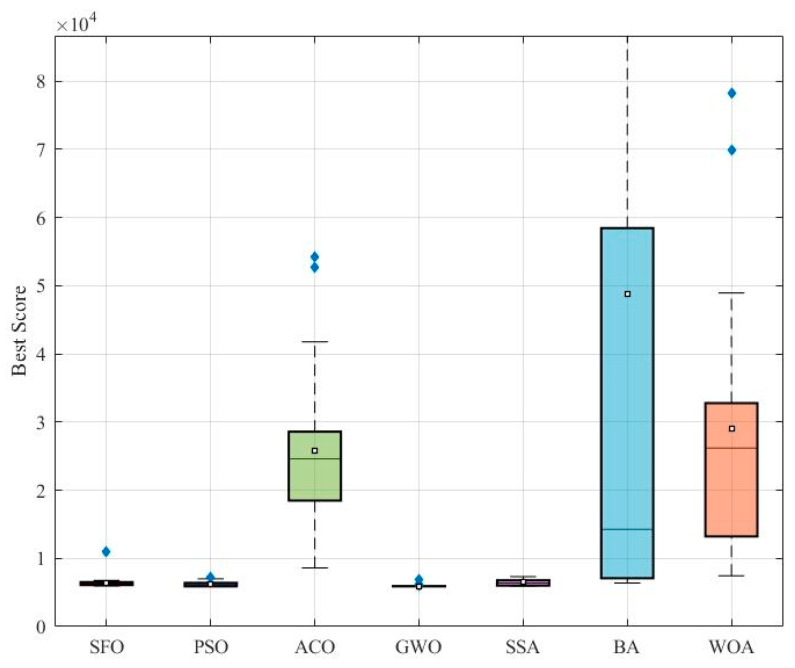
Boxplot of optimization results for the pressure vessel problem.

Figure 7 shows the boxplot of the best fitness values achieved by each algorithm. While SFO displays a relatively concentrated distribution with few outliers, it is slightly outperformed by GWO and PSO in terms of median and overall spread. Notably, GWO achieves the smallest variation, indicating highly stable performance, whereas BA and WOA exhibit much larger dispersion.

**Figure 8:**
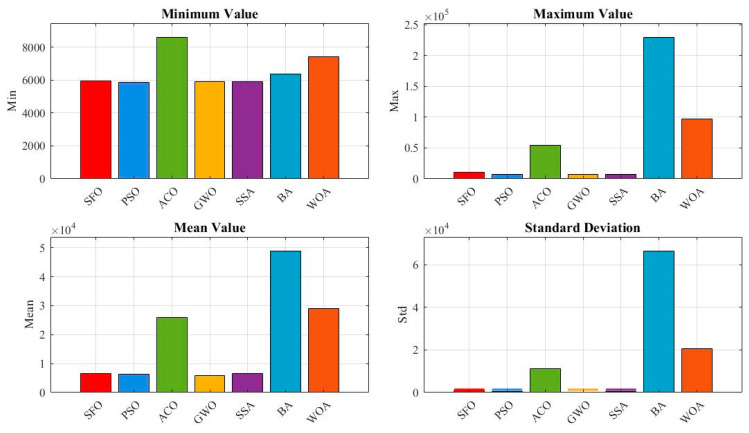
Comparison of statistical indicators for the pressure vessel design optimization.

Figure 8 provides a breakdown of four statistical metrics: minimum, maximum, mean, and standard deviation. SFO achieves competitive results, with moderate mean and variance values, but trails behind GWO and PSO, both of which demonstrate lower mean and standard deviation, suggesting better convergence precision and consistency.

Table 5 lists the best solutions found by each algorithm. SFO’s best-found solution yields a relatively low objective value, though not the lowest. PSO obtains a slightly better optimum value, while ACO and WOA show significantly higher costs.

Table 6 summarizes the statistical indicators and includes the Friedman ranking and Wilcoxon signed-rank test. SFO ranks third overall (F-Rank = 3), following GWO (Rank 1) and PSO (Rank 2). According to the Wilcoxon test, SFO is statistically superior to several weaker algorithms (e.g., ACO, BA, WOA), but not significantly better than GWO and PSO (*p* > 0.05 in these cases).

In summary, although SFO does not outperform all competitors on this specific problem, it achieves stable and competitive performance and remains statistically better than several mainstream algorithms. This suggests that SFO is a reliable optimization approach, though further tuning or hybridization may be needed for pressure vessel design task with strong structural constraints.

### 4.2. Speed Reducer Design

**Figure 9:**
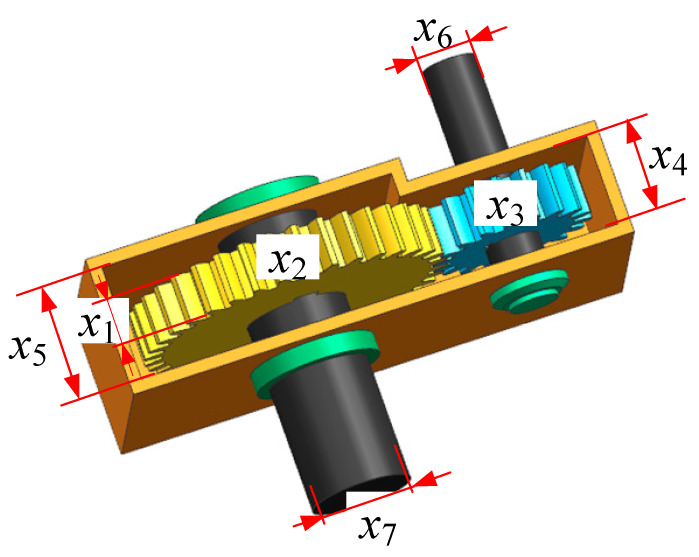
Schematic diagram of the speed reducer design problem.

The speed reducer design problem is a classical constrained engineering optimization problem. The objective of this problem is to minimize the total weight of the speed reducer. As illustrated in Figure 9, the speed reducer consists of two independent shafts equipped with gears, which are sequentially connected to the main shaft via bearings. The optimization problem involves seven design variables and eleven inequality constraints. The decision variables include the following: face width (x_1_), module of the teeth (x_2_), number of teeth on the pinion (x_3_), length of the first shaft between bearings (x_4_), length of the second shaft between bearings (x_5_), diameter of the first shaft (x_6_), and diameter of the second shaft (x_7_).

The detailed mathematical formulation is provided below:

consider: $x=(x_{1},x_{2},x_{3},x_{4},x_{5},x_{6},x_{7})$

Minimize:

(13) $$\begin{array}{c} f(x)=0.7854x_{1}x_{2}^{2}(3.3333x_{3}^{2}+14.9334x_{3}-43.0934)-1.508x_{1}(x_{7}^{2}+x_{6}^{2})\\ \;\;+0.7854(x_{4}x_{6}^{2}+x_{5}x_{7}^{2})+7.477(x_{6}^{3}+x_{7}^{3}) \end{array}$$

Subject to:

$$\left\{\begin{array}{l} g_{1}(x)=-x_{1}x_{2}^{2}x_{3}+27\leq 0\\ g_{2}(x)=-x_{1}x_{2}^{2}x_{3}^{2}+397.5\leq 0\\ g_{3}(x)=-x_{2}x_{6}^{4}x_{3}x_{4}^{-3}+1.93\leq 0\\ g_{4}(x)=-x_{2}x_{7}^{4}x_{3}x_{5}^{-3}+1.93\leq 0\\ g_{5}(x)=10x_{6}^{-3}\sqrt{16.91\times 10^{6}+{\left(745x_{4}x_{2}^{-1}x_{3}^{-1}\right)}^{2}}-1100\leq 0\\ g_{6}(x)=10x_{7}^{-3}\sqrt{157.5\times 10^{6}+{\left(745x_{5}x_{2}^{-1}x_{3}^{-1}\right)}^{2}}-850\leq 0\\ g_{7}(x)=x_{2}x_{3}-40\leq 0\\ g_{8}(x)=-x_{1}x_{2}^{-1}+5\leq 0\\ g_{9}(x)=x_{1}x_{2}^{-1}-12\leq 0\\ g_{10}(x)=1.5x_{6}-x_{4}+1.9\leq 0\\ g_{11}(x)=1.1x_{7}-x_{5}+1.9\leq 0 \end{array}\right.$$

Bounds:

$$\left\{\begin{array}{l} 2.6\leq x_{1}\leq 3.6;0.7\leq x_{2}\leq 0.8;17\leq x_{3}\leq 28\\ 7.3\leq x_{4};x_{5}\leq 8.3;2.9\leq x_{6}\leq 3.9;5\leq x_{7}\leq 5.5 \end{array}\right.$$

To evaluate the performance of the proposed SFO, we applied it to the classic speed reducer design problem and compared it with six widely used metaheuristic algorithms: PSO, ACO, GWO, SSA, BA, and WOA. The comparative analysis was conducted from multiple perspectives, including solution distribution, statistical indicators, and significance tests.

**Figure 10:**
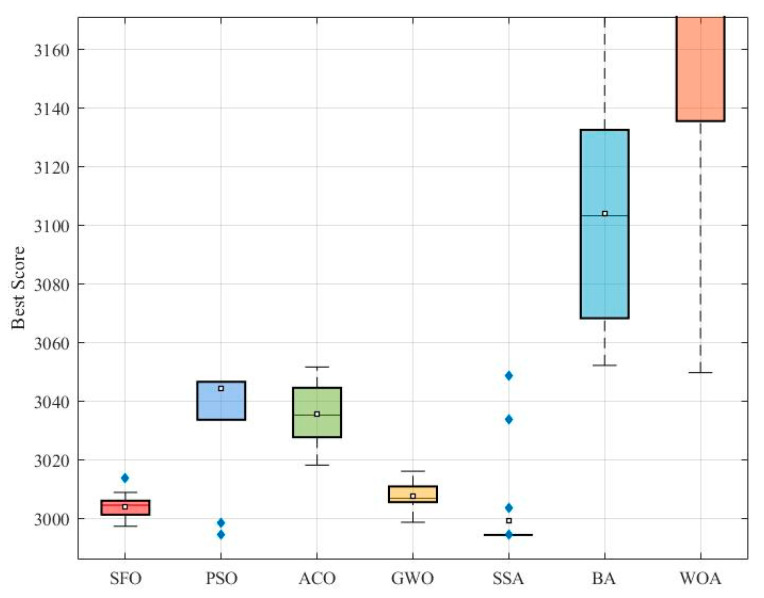
Comparative boxplot of algorithms for the speed reducer design.

Figure 10 presents a boxplot of the best objective values obtained by each algorithm over 50 independent runs. It can be observed that SSA achieves the lowest median and overall tighter spread, indicating its superior consistency and performance. The SFO algorithm shows a relatively narrow interquartile range and low median value, reflecting stable performance and outperforming PSO, ACO, GWO, BA, and WOA.

**Figure 11:**
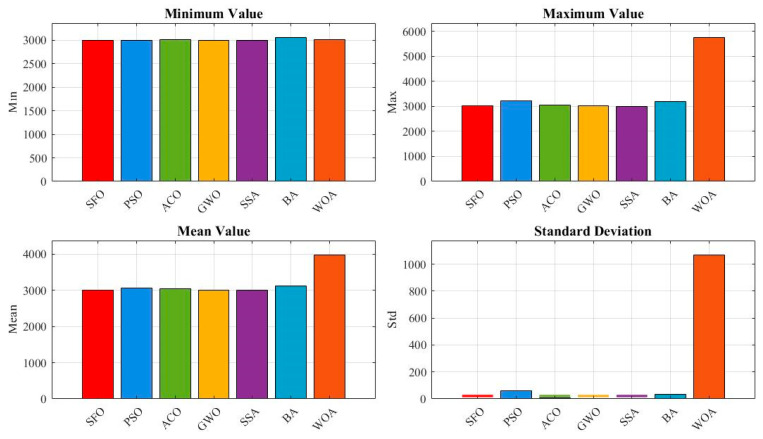
Evaluation metrics of optimization algorithms for the speed reducer design.

Figure 11 provides a bar chart comparison of four statistical indicators: minimum, maximum, mean, and standard deviation. SFO ranks second in minimum and mean values, slightly behind SSA, which achieves the best scores across all metrics. The standard deviation of SFO is notably lower than that of most compared algorithms, demonstrating robust convergence.

Table 7 lists the best design variables and corresponding objective values found by each algorithm. The best solution obtained by SFO is f(x) = 3006.9523, which is only slightly higher than SSA’s f(x) = 2994.4711, reinforcing the conclusion that SFO is a strong competitor but marginally outperformed by SSA. Nonetheless, SFO yields better solutions than PSO, ACO, GWO, BA, and WOA in this context.

Table 8 presents the statistical comparison results, including Min, Max, Mean, Std, Friedman ranking (F-Rank), Wilcoxon test outcomes, and corresponding *p*-values. SFO obtains a Friedman Rank of 2, second only to SSA, which ranks first. The Wilcoxon test confirms that the performance difference between SFO and the weaker algorithms (e.g., WOA, BA, and ACO) is statistically significant at the 0.05 level. Although SSA surpasses SFO in terms of optimality, the performance of SFO is statistically superior to most other algorithms.

Overall, SFO demonstrates strong optimization capabilities on the speed reducer design problem. While SSA exhibits the best overall performance, SFO consistently outperforms PSO, ACO, GWO, BA, and WOA across most metrics, showing its effectiveness and stability.

### 4.3. Gear Train Design Problem

**Figure 12:**
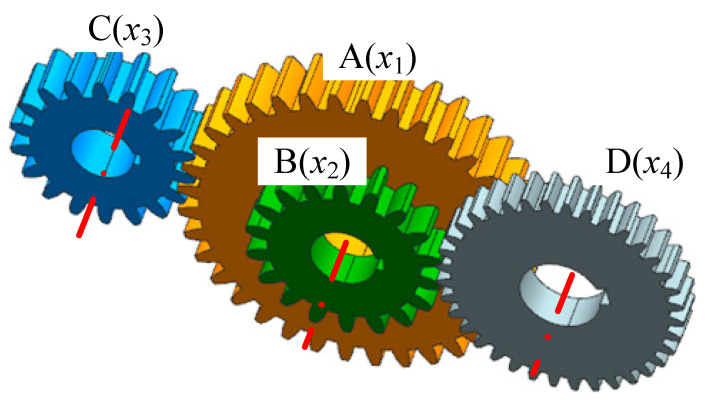
Schematic diagram of the gear train design problem.

The gear train design problem is a typical unconstrained discrete integer optimization problem in mechanical engineering. The objective is to minimize the gear ratio error between the input and output shafts. The gear ratio is defined as the ratio of the angular velocity of the output shaft to that of the input shaft. Let the number of teeth on gears A, B, C, and D be denoted as *x*_1_, *x*_2_, *x*_3_, and *x*_4_, respectively, as shown in Figure 12.

The mathematical model of the optimization problem is formulated as follows:

consider: $x=[\begin{array}{cccc} x_{1} & x_{2} & x_{3} & x_{4} \end{array}]$

Minimize:

(14) $$f(x)={\left(\frac{1}{6.931}-\frac{x_{1}x_{2}}{x_{3}x_{4}}\right)}^{2}$$

Subject to: $12\leq x_{1},x_{2},x_{3},x_{4}\leq 60,x_{1},x_{2},x_{3},x_{4}\in \mathbb{Z}$

To further evaluate the effectiveness of the proposed SFO, this section investigates its performance on the gear train design problem, a discrete, integer, and nonlinear optimization task in mechanical systems. The goal is to minimize the deviation from a target gear ratio. SFO is compared with six established metaheuristic algorithms: PSO, ACO, GWO, SSA, BA, and WOA.

**Figure 13:**
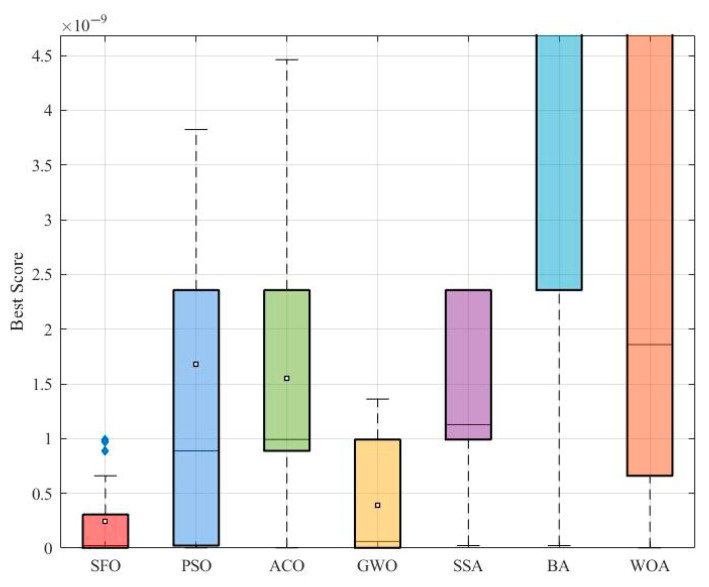
Comparative boxplot of algorithms for the gear train design problem.

As shown in Figure 13, the boxplot reveals the distribution of the best objective values over 50 independent runs. The SFO algorithm clearly outperforms all other algorithms, achieving the lowest median value and the narrowest spread. In contrast, algorithms such as BA and WOA exhibit much higher variability and worse overall solution quality, with significant outliers. This indicates that SFO not only finds better solutions but also maintains high stability and robustness.

**Figure 14:**
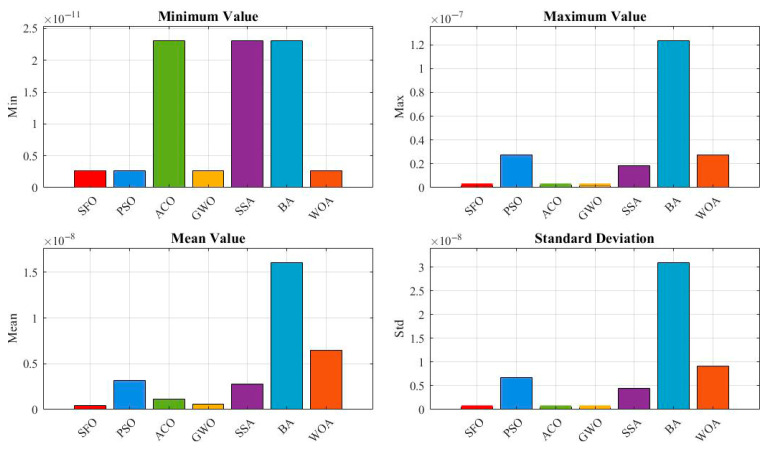
Evaluation metrics of the algorithms for the gear train design problem.

Figure 14 provides a quantitative comparison in terms of minimum, maximum, mean, and standard deviation values. SFO achieves the lowest mean and standard deviation, highlighting its consistent convergence toward the global optimum. Notably, although all algorithms reach a best fitness of 0 (as shown in Table 7), the statistical indicators in this figure show that only SFO consistently converges to zero with minimal variance, while other algorithms exhibit higher average errors and wider dispersions.

Table 9 lists the best gear tooth combinations and corresponding objective values for each algorithm. All algorithms achieve an objective value of 0 at least once, demonstrating the solvability of the problem. However, the gear combinations identified by SFO—(53,30,13,51)—represent one of the ideal configurations that precisely meet the desired gear ratio. The robustness of SFO in repeatedly finding such solutions is further confirmed by the statistical results in Table 8.

Table 10 presents the statistical analysis of algorithm performance, including minimum, maximum, mean, standard deviation, Friedman ranking (F-Rank), Wilcoxon signed-rank test, and *p*-values. SFO ranks first (F-Rank = 1), with a mean value of 4.491 × 10^−10^ and the smallest standard deviation of 4.848 × 10^−10^. The Wilcoxon test confirms that the performance of SFO is statistically superior to all other competitors at the 0.05 significance level. These results strongly support the effectiveness and reliability of SFO for discrete, nonlinear problems such as gear train design.

Across the three engineering design problems investigated—pressure vessel design, speed reducer design, and gear train design—the proposed SFO consistently demonstrates competitive optimization performance. Although SFO is slightly outperformed by GWO and PSO in the pressure vessel case, it achieves stable convergence and ranks among the top three algorithms. In the speed reducer problem, SFO exhibits superior performance over five of the six compared algorithms, trailing only SSA by a narrow margin. Most notably, in the gear train design problem, SFO ranks first in all statistical indicators, clearly outperforming all competitors in both accuracy and robustness. These results collectively highlight the generalizability, reliability, and strong optimization capability of SFO across a diverse set of engineering scenarios, including both continuous and discrete design domains.

These engineering cases, together with the preceding CEC2022 benchmark tests, demonstrate that SFO is not limited to specific types of problems but exhibits strong versatility across diverse optimization scenarios. It performs reliably on both numerical and real-world design problems, including continuous and discrete domains, unimodal and multimodal landscapes, and problems with complex structural constraints. While no algorithm can guarantee superiority in all possible cases—as asserted by the No-Free-Lunch (NFL) theorem—the experimental results confirm that SFO is a robust and competitive alternative for solving complex engineering and computational optimization tasks.

## 5. Conclusions

This paper presents the sharpbelly fish optimizer (SFO), a novel bio-inspired metaheuristic algorithm designed based on the ecological behaviors of Hemiculter leucisculus. By integrating four adaptive behaviors—fast swimming, swarm gathering, stagnation-triggered dispersal, and escape under disturbance—SFO achieves an effective balance between global exploration and local exploitation. The main contributions and findings are summarized as follows:

(1) SFO introduces a biologically driven search strategy that combines fitness-based motion and population diversity regulation. The algorithm is computationally efficient, with linear time complexity in terms of population size, problem dimension, and iteration count.

(2) On the CEC2022 benchmark suite, SFO demonstrates competitive or superior convergence accuracy, speed, and robustness compared to several state-of-the-art algorithms, including PSO, ACO, GWO, SSA, and others.

(3) SFO has been validated on three representative engineering design problems, covering both continuous and discrete domains. It consistently achieves top-level performance across multiple evaluation metrics, confirming its strong adaptability and reliability in practical engineering scenarios.

These results confirm that SFO is a versatile and effective optimization tool, capable of solving complex real-world problems with various structural characteristics and constraints.

While the SFO algorithm demonstrates strong convergence speed, stability, and adaptability across various benchmark functions, it still has certain limitations. For instance, its performance may degrade in extremely high-dimensional or highly deceptive multimodal landscapes, where more sophisticated memory-based or adaptive mechanisms may be required. Nevertheless, its simple structure, ease of implementation, and competitive performance make it a promising candidate for addressing a wide range of practical optimization problems in engineering and industrial applications.

Future work will focus on enhancing SFO through hybridization with neural or evolutionary mechanisms, extending it to multi-objective, dynamic, or multimodal scenarios, and exploring broader applications such as intelligent scheduling and automated industrial design.

## Figures and Tables

**Table 1:** Comparison between existing metaheuristic algorithms and the proposed SFO.

Algorithms	Inspiration	Advantages	Limitations
SSA [11]	Sparrow foraging.	Adaptive roles; strong convergence.	Sensitive to parameters.
MRFO [13]	Manta ray foraging.	Chain-based search; diverse movement.	Lacks adaptive exploitation.
EO [14]	Equilibrium states.	Parameter-free; wide exploration.	Exploitation capacity is limited.
RUN [15]	Randomized unified operators.	Operator diversity; good performance on CEC.	Instability in high dimensions.
HGS [16]	Genetic hybridization.	Robust for constrained optimization.	Complex structure.
AHA [17]	Algae hunting and aggregation.	Strong adaptability; biological realism.	Risk of slow convergence.
DBO [18]	Dung beetle behavior.	Multi-strategy; discrete-continuous hybrid.	May stagnate in flat regions.
GSFO [19]	Goose migration and foraging.	Effective migration-based exploitation.	Weak in highly multimodal tasks.
HHO [20]	Harris hawk variants.	Phase-switching strategies.	Complex control parameters.
AOA [21]	Arithmetic optimization.	Mathematical modeling; simple.	Risk of early convergence.
CMA [22]	Cuckoo mimicry adaptation.	Mimicry-guided exploration.	Lacks convergence stability.
MBO [23]	Magpie brood parasitism.	Long-jump escape mechanism.	Weak convergence in fine-tuning.
Proposed SFO	Sharpbelly fish behavior.	Multi-behavior synergy; adaptive jump and gathering.	Requires tuning for strict constraints.

**Table 2:** Summary of CEC2022 benchmark functions used in this study.

No.	Function Name	Category	*f_i_* *
F1	Shifted and full Rotated Zakharov Function	Unimodal Function	300
F2	Shifted and full Rotated Rosenbrock’s Function	Unimodal Function	400
F3	Shifted and full Rotated Expanded Schaffer’s F6 Function	Basic Function	600
F4	Shifted and full Rotated Non-Continuous Rastrigin’s Function	Basic Function	800
F5	Shifted and full Rotated Levy Function	Basic Function	900
F6	Hybrid Function 1 (*N* = 3)	Hybrid Function	1800
F7	Hybrid Function 2 (*N* = 6)	Hybrid Function	2000
F8	Hybrid Function 3 (*N* = 5)	Hybrid Function	2200
F9	Composition Function 1 (*N* = 5)	Composition Function	2300
F10	Composition Function 2 (*N* = 4)	Composition Function	2400
F11	Composition Function 3 (*N* = 5)	Composition Function	2600
F12	Composition Function 4 (*N* = 6)	Composition Function	2700

Search range: [−100, 100]*^D^*; Source: adapted from the CEC2022 technical report.

**Table 3:** Control Parameter Settings of All Compared Algorithms.

Algorithms	Parameter Settings
PSO	*c*_1_ = 1.5, *c*_2_ = 1.5, *W_max_* = 0.9, *W_min_* = 0.4
ACO	*rho* = 0.9, *P*_0_ = 0.2, step = 0.1
GA	*pc* = 0.8, *pm* = 0.1, elite_ratio = 0.05
GWO	*r*_1_ = [0, 1], *r*_2_ = [0, 1]
SSA	PD = 0.2, SD = 0.1, ST = 0.8
BA	A = 0.6·ones(N,1), r0 = 0.7, Af = 0.9, Rf = 0.9
WOA	*r*_1_ = [0, 1], *r*_2_ = [0, 1]
SFO	α = 0.3, δ = 0.05·(ub-lb), Ts = 5, Pf = 0.4

**Table 4:** Comparative performance on CEC2022 under different dimensions.

Functions	Algorithms	Dim = 10			Dim = 20		
		Max	Mean	Std.	Max	Mean	Std.
F1	SFO	2.18 × 10^3^	5.43 × 10^2^	3.80 × 10^2^	1.21 × 10^4^	6.21 × 10^3^	2.79 × 10^3^
	PSO	3.17 × 10^2^	3.02 × 10^2^	5.07 × 10^0^	3.09 × 10^4^	7.42 × 10^3^	5.72 × 10^3^
	ACO	1.24 × 10^4^	8.99 × 10^3^	1.87 × 10^3^	6.15 × 10^4^	3.75 × 10^4^	8.44 × 10^3^
	GA	1.13 × 10^6^	7.81 × 10^4^	2.02 × 10^5^	6.70 × 10^7^	2.48 × 10^6^	1.22 × 10^7^
	GWO	7.52 × 10^3^	2.48 × 10^3^	2.14 × 10^3^	1.92 × 10^4^	1.22 × 10^4^	3.99 × 10^3^
	SSA	4.72 × 10^2^	3.13 × 10^2^	3.26 × 10^1^	8.29 × 10^3^	2.45 × 10^3^	1.61 × 10^3^
	BA	1.51 × 10^4^	6.56 × 10^3^	4.15 × 10^3^	9.90 × 10^4^	4.82 × 10^4^	1.98 × 10^4^
	WOA	5.08 × 10^4^	1.31 × 10^4^	8.74 × 10^3^	1.51 × 10^5^	5.58 × 10^4^	3.14 × 10^4^
F2	SFO	4.96 × 10^2^	4.25 × 10^2^	2.65 × 10^1^	7.22 × 10^2^	5.58 × 10^2^	6.84 × 10^1^
	PSO	8.58 × 10^2^	4.47 × 10^2^	9.92 × 10^1^	6.09 × 10^2^	4.92 × 10^2^	4.35 × 10^1^
	ACO	5.89 × 10^2^	5.18 × 10^2^	3.27 × 10^1^	1.88 × 10^3^	1.34 × 10^3^	2.24 × 10^2^
	GA	1.72 × 10^3^	9.91 × 10^2^	2.88 × 10^2^	3.41 × 10^3^	1.69 × 10^3^	6.02 × 10^2^
	GWO	4.95 × 10^2^	4.21 × 10^2^	2.39 × 10^1^	6.48 × 10^2^	5.07 × 10^2^	5.03 × 10^1^
	SSA	4.75 × 10^2^	4.18 × 10^2^	2.26 × 10^1^	6.07 × 10^2^	4.77 × 10^2^	3.88 × 10^1^
	BA	5.69 × 10^2^	4.50 × 10^2^	4.05 × 10^1^	8.54 × 10^2^	6.05 × 10^2^	8.28 × 10^1^
	WOA	1.73 × 10^3^	6.46 × 10^2^	2.59 × 10^2^	2.89 × 10^3^	1.50 × 10^3^	5.12 × 10^2^
F3	SFO	6.33 × 10^2^	6.19 × 10^2^	6.33 × 10^0^	6.76 × 10^2^	6.39 × 10^2^	1.14 × 10^1^
	PSO	6.44 × 10^2^	6.14 × 10^2^	9.61 × 10^0^	6.77 × 10^2^	6.39 × 10^2^	1.46 × 10^1^
	ACO	6.48 × 10^2^	6.38 × 10^2^	4.77 × 10^0^	6.81 × 10^2^	6.66 × 10^2^	8.30 × 10^0^
	GA	6.72 × 10^2^	6.48 × 10^2^	9.99 × 10^0^	7.13 × 10^2^	6.82 × 10^2^	1.25 × 10^1^
	GWO	6.03 × 10^2^	6.01 × 10^2^	1.06 × 10^0^	6.14 × 10^2^	6.06 × 10^2^	3.45 × 10^0^
	SSA	6.55 × 10^2^	6.16 × 10^2^	1.18 × 10^1^	6.74 × 10^2^	6.53 × 10^2^	1.41 × 10^1^
	BA	6.76 × 10^2^	6.55 × 10^2^	1.24 × 10^1^	7.06 × 10^2^	6.77 × 10^2^	1.37 × 10^1^
	WOA	6.65 × 10^2^	6.42 × 10^2^	1.12 × 10^1^	6.89 × 10^2^	6.75 × 10^2^	9.02 × 10^0^
F4	SFO	8.54 × 10^2^	8.46 × 10^2^	5.64 × 10^0^	9.80 × 10^2^	9.54 × 10^2^	1.41 × 10^1^
	PSO	8.49 × 10^2^	8.24 × 10^2^	9.38 × 10^0^	9.26 × 10^2^	8.73 × 10^2^	2.31 × 10^1^
	ACO	8.77 × 10^2^	8.64 × 10^2^	8.00 × 10^0^	1.02 × 10^3^	9.90 × 10^2^	1.20 × 10^1^
	GA	8.88 × 10^2^	8.50 × 10^2^	1.18 × 10^1^	1.03 × 10^3^	9.81 × 10^2^	1.93 × 10^1^
	GWO	8.34 × 10^2^	8.15 × 10^2^	6.56 × 10^0^	9.21 × 10^2^	8.59 × 10^2^	2.59 × 10^1^
	SSA	8.81 × 10^2^	8.34 × 10^2^	1.79 × 10^1^	9.74 × 10^2^	9.06 × 10^2^	2.77 × 10^1^
	BA	8.88 × 10^2^	8.54 × 10^2^	1.83 × 10^1^	1.01 × 10^3^	9.50 × 10^2^	3.86 × 10^1^
	WOA	8.83 × 10^2^	8.49 × 10^2^	1.08 × 10^1^	1.00 × 10^3^	9.61 × 10^2^	2.34 × 10^1^
F5	SFO	1.12 × 10^3^	9.87 × 10^2^	5.28 × 10^1^	3.26 × 10^3^	2.00 × 10^3^	5.39 × 10^2^
	PSO	1.04 × 10^3^	9.35 × 10^2^	3.61 × 10^1^	3.33 × 10^3^	1.78 × 10^3^	5.79 × 10^2^
	ACO	2.11 × 10^3^	1.80 × 10^3^	2.07 × 10^2^	7.39 × 10^3^	5.91 × 10^3^	6.88 × 10^2^
	GA	1.52 × 10^3^	1.19 × 10^3^	1.60 × 10^2^	3.20 × 10^3^	2.41 × 10^3^	4.47 × 10^2^
	GWO	9.59 × 10^2^	9.11 × 10^2^	1.84 × 10^1^	1.65 × 10^3^	1.09 × 10^3^	1.63 × 10^2^
	SSA	1.89 × 10^3^	1.20 × 10^3^	2.53 × 10^2^	4.07 × 10^3^	2.77 × 10^3^	6.16 × 10^2^
	BA	3.88 × 10^3^	2.49 × 10^3^	7.24 × 10^2^	8.08 × 10^3^	5.24 × 10^3^	1.45 × 10^3^
	WOA	2.01 × 10^3^	1.51 × 10^3^	2.48 × 10^2^	5.57 × 10^3^	3.53 × 10^3^	7.46 × 10^2^
F6	SFO	2.49 × 10^5^	2.13 × 10^4^	4.82 × 10^4^	3.32 × 10^6^	5.47 × 10^5^	8.02 × 10^5^
	PSO	8.18 × 10^3^	3.88 × 10^3^	2.88 × 10^3^	2.06 × 10^4^	4.87 × 10^3^	3.70 × 10^3^
	ACO	2.04 × 10^7^	9.05 × 10^6^	5.66 × 10^6^	1.40 × 10^9^	5.11 × 10^8^	3.70 × 10^8^
	GA	5.27 × 10^8^	7.62 × 10^7^	1.25 × 10^8^	4.92 × 10^9^	1.29 × 10^9^	1.23 × 10^9^
	GWO	8.27 × 10^3^	5.13 × 10^3^	2.33 × 10^3^	1.26 × 10^7^	7.48 × 10^5^	2.52 × 10^6^
	SSA	8.09 × 10^3^	3.66 × 10^3^	1.92 × 10^3^	2.51 × 10^4^	8.80 × 10^3^	7.35 × 10^3^
	BA	2.15 × 10^4^	1.23 × 10^4^	4.78 × 10^3^	7.38 × 10^5^	3.57 × 10^5^	1.36 × 10^5^
	WOA	2.19 × 10^8^	1.59 × 10^7^	5.54 × 10^7^	4.52 × 10^9^	9.73 × 10^8^	1.11 × 10^9^
F7	SFO	2.08 × 10^3^	2.06 × 10^3^	7.99 × 10^0^	2.20 × 10^3^	2.13 × 10^3^	2.23 × 10^1^
	PSO	2.10 × 10^3^	2.05 × 10^3^	1.86 × 10^1^	2.27 × 10^3^	2.15 × 10^3^	5.07 × 10^1^
	ACO	2.11 × 10^3^	2.08 × 10^3^	1.37 × 10^1^	2.25 × 10^3^	2.19 × 10^3^	2.21 × 10^1^
	GA	2.20 × 10^3^	2.11 × 10^3^	3.71 × 10^1^	2.38 × 10^3^	2.23 × 10^3^	7.25 × 10^1^
	GWO	2.07 × 10^3^	2.03 × 10^3^	1.36 × 10^1^	2.27 × 10^3^	2.09 × 10^3^	5.02 × 10^1^
	SSA	2.08 × 10^3^	2.04 × 10^3^	1.49 × 10^1^	2.33 × 10^3^	2.17 × 10^3^	6.56 × 10^1^
	BA	2.21 × 10^3^	2.11 × 10^3^	3.56 × 10^1^	2.42 × 10^3^	2.24 × 10^3^	8.29 × 10^1^
	WOA	2.16 × 10^3^	2.09 × 10^3^	3.22 × 10^1^	2.39 × 10^3^	2.22 × 10^3^	7.23 × 10^1^
F8	SFO	2.35 × 10^3^	2.24 × 10^3^	3.05 × 10^1^	2.39 × 10^3^	2.29 × 10^3^	5.61 × 10^1^
	PSO	2.36 × 10^3^	2.24 × 10^3^	3.11 × 10^1^	2.38 × 10^3^	2.28 × 10^3^	6.11 × 10^1^
	ACO	2.25 × 10^3^	2.24 × 10^3^	4.36 × 10^0^	2.57 × 10^3^	2.39 × 10^3^	6.32 × 10^1^
	GA	2.40 × 10^3^	2.27 × 10^3^	6.00 × 10^1^	6.12 × 10^3^	2.83 × 10^3^	9.69 × 10^2^
	GWO	2.23 × 10^3^	2.22 × 10^3^	6.03 × 10^0^	2.35 × 10^3^	2.26 × 10^3^	5.05 × 10^1^
	SSA	2.24 × 10^3^	2.23 × 10^3^	4.96 × 10^0^	2.35 × 10^3^	2.25 × 10^3^	2.20 × 10^1^
	BA	2.63 × 10^3^	2.32 × 10^3^	1.35 × 10^2^	2.99 × 10^3^	2.58 × 10^3^	1.90 × 10^2^
	WOA	2.29 × 10^3^	2.24 × 10^3^	1.72 × 10^1^	2.50 × 10^3^	2.31 × 10^3^	7.94 × 10^1^
F9	SFO	2.57 × 10^3^	2.55 × 10^3^	8.36 × 10^0^	2.73 × 10^3^	2.59 × 10^3^	6.33 × 10^1^
	PSO	2.68 × 10^3^	2.55 × 10^3^	4.32 × 10^1^	2.71 × 10^3^	2.54 × 10^3^	5.21 × 10^1^
	ACO	2.73 × 10^3^	2.60 × 10^3^	4.99 × 10^1^	2.94 × 10^3^	2.76 × 10^3^	7.79 × 10^1^
	GA	2.98 × 10^3^	2.78 × 10^3^	7.80 × 10^1^	4.15 × 10^3^	3.33 × 10^3^	2.90 × 10^2^
	GWO	2.64 × 10^3^	2.57 × 10^3^	3.38 × 10^1^	2.63 × 10^3^	2.51 × 10^3^	2.82 × 10^1^
	SSA	2.68 × 10^3^	2.54 × 10^3^	4.49 × 10^1^	2.51 × 10^3^	2.49 × 10^3^	5.04 × 10^0^
	BA	2.72 × 10^3^	2.63 × 10^3^	4.19 × 10^1^	2.85 × 10^3^	2.65 × 10^3^	7.53 × 10^1^
	WOA	2.78 × 10^3^	2.69 × 10^3^	5.86 × 10^1^	3.24 × 10^3^	2.86 × 10^3^	1.42 × 10^2^
F10	SFO	2.66 × 10^3^	2.57 × 10^3^	7.58 × 10^1^	6.89 × 10^3^	4.28 × 10^3^	1.96 × 10^3^
	PSO	2.65 × 10^3^	2.55 × 10^3^	6.38 × 10^1^	5.35 × 10^3^	4.11 × 10^3^	9.34 × 10^2^
	ACO	3.80 × 10^3^	2.57 × 10^3^	2.38 × 10^2^	6.98 × 10^3^	5.31 × 10^3^	1.85 × 10^3^
	GA	4.02 × 10^3^	2.80 × 10^3^	3.48 × 10^2^	7.94 × 10^3^	6.73 × 10^3^	8.42 × 10^2^
	GWO	3.00 × 10^3^	2.59 × 10^3^	9.60 × 10^1^	6.52 × 10^3^	3.57 × 10^3^	8.76 × 10^2^
	SSA	3.51 × 10^3^	2.60 × 10^3^	2.02 × 10^2^	5.88 × 10^3^	3.54 × 10^3^	9.09 × 10^2^
	BA	4.33 × 10^3^	2.83 × 10^3^	5.06 × 10^2^	7.24 × 10^3^	5.24 × 10^3^	9.31 × 10^2^
	WOA	4.65 × 10^3^	2.76 × 10^3^	4.25 × 10^2^	7.84 × 10^3^	6.03 × 10^3^	1.31 × 10^3^
F11	SFO	4.04 × 10^3^	3.08 × 10^3^	2.18 × 10^2^	4.80 × 10^3^	3.74 × 10^3^	3.85 × 10^2^
	PSO	3.77 × 10^3^	2.95 × 10^3^	1.68 × 10^2^	5.44 × 10^3^	4.17 × 10^3^	4.54 × 10^2^
	ACO	3.27 × 10^4^	2.43 × 10^4^	4.73 × 10^3^	1.40 × 10^5^	1.13 × 10^5^	1.27 × 10^4^
	GA	2.22 × 10^4^	1.35 × 10^4^	4.20 × 10^3^	7.01 × 10^4^	3.87 × 10^4^	1.18 × 10^4^
	GWO	3.36 × 10^3^	2.99 × 10^3^	1.39 × 10^2^	5.07 × 10^3^	3.77 × 10^3^	5.14 × 10^2^
	SSA	2.95 × 10^3^	2.86 × 10^3^	1.07 × 10^2^	3.23 × 10^3^	2.97 × 10^3^	6.59 × 10^1^
	BA	5.45 × 10^4^	2.54 × 10^4^	1.09 × 10^4^	1.87 × 10^5^	9.64 × 10^4^	3.16 × 10^4^
	WOA	5.11 × 10^3^	3.89 × 10^3^	6.61 × 10^2^	9.97 × 10^3^	8.79 × 10^3^	7.49 × 10^2^
F12	SFO	2.87 × 10^3^	2.87 × 10^3^	1.07 × 10^0^	3.09 × 10^3^	3.03 × 10^3^	2.66 × 10^1^
	PSO	2.94 × 10^3^	2.88 × 10^3^	2.24 × 10^1^	3.27 × 10^3^	3.07 × 10^3^	7.42 × 10^1^
	ACO	2.87 × 10^3^	2.87 × 10^3^	1.15 × 10^0^	3.02 × 10^3^	3.00 × 10^3^	1.12 × 10^1^
	GA	3.39 × 10^3^	3.13 × 10^3^	1.09 × 10^2^	5.30 × 10^3^	4.11 × 10^3^	3.62 × 10^2^
	GWO	2.91 × 10^3^	2.87 × 10^3^	1.27 × 10^1^	3.03 × 10^3^	2.98 × 10^3^	2.51 × 10^1^
	SSA	2.88 × 10^3^	2.87 × 10^3^	3.10 × 10^0^	3.21 × 10^3^	3.01 × 10^3^	5.75 × 10^1^
	BA	3.07 × 10^3^	2.97 × 10^3^	5.79 × 10^1^	3.76 × 10^3^	3.49 × 10^3^	1.86 × 10^2^
	WOA	3.08 × 10^3^	2.95 × 10^3^	5.50 × 10^1^	3.96 × 10^3^	3.35 × 10^3^	2.53 × 10^2^

**Table 5:** Comparative optimization results for the pressure vessel design.

Algorithms	x	f(x)
SFO	**[0.7806, 0.3858, 40.4269, 198.9208]**	**5900.7255**
PSO	[0.8454, 0.4179, 43.8031, 156.5988]	6010.4694
ACO	[1.1951, 1.5306, 40.6615, 199.9997]	12,605.6616
GWO	[0.7902, 0.4090, 40.9168, 191.8839]	5965.0748
SSA	[0.9957, 0.4922, 51.5914, 86.2001]	6370.8352
BA	[1.1449, 0.5518, 57.5837, 162.2747]	12,083.3905
WOA	[0.9670, 6.6654, 50.0317, 98.0947]	33,839.7347

**Table 6:** Comparative results and significance analysis for the pressure vessel optimization.

Algorithms	Min	Max	Mean	Std.	F-Rank	Wilcoxon	*p*-Value
SFO	5.94 × 10^3^	1.09 × 10^4^	6.45 × 10^3^	8.80 × 10^2^	3	—	—
PSO	5.89 × 10^3^	7.32 × 10^3^	6.25 × 10^3^	4.18 × 10^2^	2	(+)	8.77 × 10^−2^
ACO	8.61 × 10^3^	5.43 × 10^4^	2.58 × 10^4^	1.13 × 10^4^	6	(+)	3.69 × 10^−11^
GWO	5.89 × 10^3^	6.84 × 10^3^	5.96 × 10^3^	1.74 × 10^2^	1	(−)	2.67 × 10^−9^
SSA	5.99 × 10^3^	7.32 × 10^3^	6.48 × 10^3^	4.73 × 10^2^	4	(+)	3.95 × 10^−1^
BA	6.38 × 10^3^	2.30 × 10^5^	4.89 × 10^4^	6.64 × 10^4^	5	(+)	7.77 × 10^−9^
WOA	7.45 × 10^3^	9.65 × 10^4^	2.90 × 10^4^	2.07 × 10^4^	7	(+)	4.50 × 10^−11^

**Table 7:** Optimization outcomes of various algorithms for the speed reducer design.

Algorithms	x	f(x)
SFO	**[3.5003, 0.7001, 17.0000, 7.9583, 7.9773, 3.3518, 5.2868]**	**3006.9523**
PSO	[3.6000, 0.7000, 17.0000, 7.3000, 7.7153, 3.3502, 5.2867]	3033.7486
ACO	[3.5243, 0.7000, 17.0015, 7.9992, 7.8261, 3.3740, 5.3263]	3044.4453
GWO	[3.5045, 0.7003, 17.0000, 7.5538, 7.9008, 3.3633, 5.2882]	3008.2493
SSA	[3.5000, 0.7000, 17.0000, 7.3000, 7.7153, 3.3502, 5.2867]	2994.4711
BA	[3.6000, 0.7000, 17.0000, 7.5435, 8.3000, 3.5863, 5.3082]	3127.1021
WOA	[3.5881, 0.7000, 17.0000, 8.0302, 8.0302, 3.8794, 5.2868]	3201.3228

**Table 8:** Evaluation and statistical testing of the algorithms on the speed reducer design.

Algorithms	Min	Max	Mean	Std.	F-Rank	Wilcoxon	*p*-Value
SFO	2.996 × 10^3^	3.010 × 10^3^	3.004 × 10^3^	3.925 × 10^0^	2	—	—
PSO	2.99 × 10^3^	3.18 × 10^3^	3.04 × 10^3^	3.10 × 10^1^	4	(+)	1.16 × 10^−7^
ACO	3.01 × 10^3^	3.06 × 10^3^	3.04 × 10^3^	1.17 × 10^1^	5	(+)	6.07 × 10^−11^
GWO	3.00 × 10^3^	3.02 × 10^3^	3.01 × 10^3^	4.45 × 10^0^	3	(+)	5.94 × 10^−2^
SSA	2.99 × 10^3^	3.03 × 10^3^	3.00 × 10^3^	1.30 × 10^1^	1	(−)	1.17 × 10^−4^
BA	3.03 × 10^3^	3.18 × 10^3^	3.11 × 10^3^	3.75 × 10^1^	6	(+)	3.02 × 10^−11^
WOA	3.06 × 10^3^	3.99 × 10^97^	2.17 × 10^96^	8.47 × 10^96^	7	(+)	3.02 × 10^−11^

**Table 9:** Optimization outcomes of various algorithms for the gear train design problem.

Algorithms	x	f(x)
SFO	**[53, 30, 13, 51]**	**0**
PSO	[39, 15, 12, 32]	0
ACO	[57, 37, 12, 54]	0
GWO	[59, 21, 15, 37]	0
SSA	[47, 13, 12, 23]	0
BA	[48, 34, 12, 59]	0
WOA	[52, 15, 26, 52]	0

**Table 10:** Evaluation and statistical testing of the algorithms on the gear train design problem.

Algorithms	Min	Max	Mean	Std.	F-Rank	Wilcoxon	*p*-Value
SFO	**2.70 × 10^−12^**	**1.263 × 10^−9^**	**4.491 × 10^−10^**	**4.848 × 10^−10^**	1	—	—
PSO	2.70 × 10^−12^	2.73 × 10^−8^	3.15 × 10^−9^	6.74 × 10^−9^	3	(+)	5.55 × 10^−4^
ACO	2.31 × 10^−11^	2.36 × 10^−9^	1.15 × 10^−9^	5.92 × 10^−10^	4	(+)	8.75 × 10^−6^
GWO	2.70 × 10^−12^	1.36 × 10^−9^	5.54 × 10^−10^	5.66 × 10^−10^	2	(+)	3.00 × 10^−1^
SSA	2.31 × 10^−11^	1.83 × 10^−8^	2.75 × 10^−9^	4.44 × 10^−9^	5	(+)	3.94 × 10^−5^
BA	2.31 × 10^−11^	1.23 × 10^−7^	1.60 × 10^−8^	3.10 × 10^−8^	7	(+)	6.24 × 10^−9^
WOA	2.70 × 10^−12^	2.73 × 10^−8^	6.47 × 10^−9^	9.08 × 10^−9^	6	(+)	2.35 × 10^−5^

## Data Availability

The data are available within the article.
